# The *ent*-15**α**-Acetoxykaur-16-en-19-oic Acid Relaxes Rat Artery Mesenteric Superior via Endothelium-Dependent and Endothelium-Independent Mechanisms

**DOI:** 10.1155/2012/472821

**Published:** 2012-12-27

**Authors:** Êurica Adélia Nogueira Ribeiro, Edla de Azevedo Herculano, Cintia Danieli Ferreira da Costa, Fabiola Fialho Furtado, Emídio Vasconcelos Leitão da-Cunha, José Maria Barbosa-Filho, Marcelo Sobral da Silva, Isac Almeida de Medeiros

**Affiliations:** ^1^Escola de Enfermagem e Farmácia, Universidade Federal de Alagoas, Cidade Universitária, Tabuleiro dos Martins, 57072-970 Maceió, AL, Brazil; ^2^Laboratório de Tecnologia Farmacêutica, Universidade Federal da Paraíba, P.O. Box 5009, 58051-970 João Pessoa, PB, Brazil; ^3^Departamento de Farmácia, CCBS, Universidade Estadual da Paraíba, 58100-000 Campina Grande, PB, Brazil

## Abstract

The objective of the study was to investigate the mechanism of the relaxant activity of the *ent*-15**α**-acetoxykaur-16-en-19-oic acid (KA-acetoxy). In rat mesenteric artery rings, KA-acetoxy induced a concentration-dependent relaxation in vessels precontracted with phenylephrine. In the absence of endothelium, the vasorelaxation was significantly shifted to the right without reduction of the maximum effect. Endothelium-dependent relaxation was significantly attenuated by pretreatment with L-NAME, an inhibitor of the NO-synthase (NOS), indomethacin, an inhibitor of the cyclooxygenase, L-NAME + indomethacin, atropine, a nonselective antagonist of the muscarinic receptors, ODQ, selective inhibitor of the guanylyl cyclase enzyme, or hydroxocobalamin, a nitric oxide scavenger. The relaxation was completely reversed in the presence of L-NAME + 1 mM L-arginine or L-arginine, an NO precursor. Diterpene-induced relaxation was not affected by TEA, a nonselective inhibitor of K+ channels. The KA-acetoxy antagonized CaCl_2_-induced contractions in a concentration-dependent manner and also inhibited an 80 mM KCl-induced contraction. The KA-acetoxy did not interfere with Ca^2+^ release from intracellular stores. The vasorelaxant induced by KA-acetoxy seems to involve the inhibition of the Ca^2+^ influx and also, at least in part, by endothelial muscarinic receptors activation, NO and PGI_2_ release.

## 1. Introduction

The use of alternative therapies, herbs, and supplements occurs at a very high rate among patients with cardiovascular disease including hypertension [1]. Hypertensive disease is a major public health problem affecting 1 billion individuals, and approximately 7.1 million deaths per year may be attributable to hypertension [2]. The treatment of this disease with plant derivatives is well reported in the literature [3].

Several medicinal plants have been largely studied and their therapeutic potential has been demonstrated in animals, aiming to provide a scientific basis for the therapeutic applications. In this context, we highlight the impact and importance of medicinal plants containing diterpenoids and their action antihypertensive. For example, *Croton zambesicus* is used in traditional medicine in Africa to treat hypertension. The diterpenes isolated from the extract of plant induced vascular relaxation and antihypertensive effect [4, 5]. The *Marrubium vulgare* is a plant used as an antihypertensive agent. The crude extract decreases systolic blood pressure in spontaneously hypertensive rats [6].

*Guatteria juruensis* Diels (Annonaceae) is a large shrub or small tree indigenous to Central and South America. Various species of the genus *Guatteria* have been used as medicines in the treatment of gonorrhea, renal calculi, and pellagra, as well as diuretic [7]. The KA-acetoxy (*ent*-15*α*-acetoxykaur-16-en-19-oic acid) is a kaurane-type diterpene, isolated from leaves of this plant.

Terpenoids constitute the largest family of natural products [8, 9] and are classified by the homologous series of a number of five carbon isoprene units in their structure: hemiterpenes C5 (1 isoprene unit), monoterpenes C10 (2 isoprene units), sesquiterpenes C15 (3 isoprene units), diterpenes C20 (4 isoprene units), and triterpenes C30 (6 isoprene units) [10].

Biological assays have shown that the diterpenes exert hypotensive and antihypertensive action. The kaurenoic acid relaxes isolated rat aorta by blocking extracellular Ca^2+^ influx blocked, activation of NO-cGMP pathway, and the opening of K^+^ channels [11, 12]. Stevioside, diterpenoid glycoside, produced vasorelaxant and hypotensive effects in rats [13, 14]. The *ent*-pimara-8(14),15-dien-19-oic acid reduces vascular resistance via the inhibition of extracellular Ca^2+^ influx and release of endothelium-derived relaxing factors [15]. The diterpene ent-8(14),15-pimaradien-3*β*-ol also induced vascular relaxation [16]. Three other ent-kaurane derivates isolated from *Oyedaea verbesinoides* (Asteraceae) induced relaxation in aortic rings [17].

Based on this premise, it is evident that the importance of studies characterizes the cardiovascular activities of kaurane-type diterpene, because these compounds can be a promising source for the discovery of numerous bioactive molecules and the development of novel antihypertensive agents [3].

In a broad pharmacological screening performed in our laboratory, we observed in isolated mesenteric superior rings of rats that KA-acetoxy produced a relaxant action on smooth muscle. The present study aimed to elucidate the mechanism of this vasorelaxant effect induced by KA-acetoxy in rat isolated mesenteric rings.

## 2. Methods

### 2.1. Plant Material

The leaves of *Guatteria juruensis* Diels (Annonaceae) were collected in August 2001 at the Mocambo Reserve, near the city of Belém, State of Pará, Brazil, and identified by the Annonaceae specialist Dr. Jorge Oliveira, from the Museu Paraense Emílio Goldi (MPEG). A voucher specimen (no. 148.677) has been deposited at the Herbarium of MPEG, Belém, Pará, Brazil.

### 2.2. Procedure for Isolation of KA-Acetoxy

**Figure 1:**
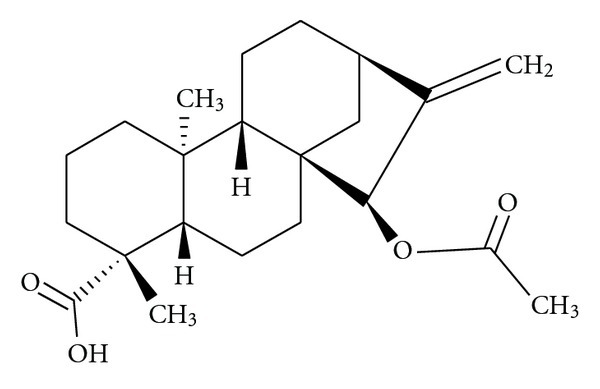
Chemical structure of KA-acetoxy.

The dried and powdered leaves (5.230 g) were extracted in a Soxhlet apparatus. The solvents used were first hexane, then chloroform, and finally EtOH. The hexane extract yielded after cooling a white precipitate. This precipitate, after filtration and washing with hot hexane, yielded KA-acetoxy (5.38 g). KA-acetoxy was identified by means of spectroscopic methods, mainly 1D and 2D NMR as *ent*-15*α*-acetoxykaur-16-en-19-oic acid [18] (Figure 1). In fact just using hot hexane, KA-acetoxy was obtained as a pure compound, according with the melting point (mp = 178–180°C) in comparison with the literature and by means of spectroscopic methods. The isolation of KA-acetoxy was previously described by [19] and its structure has been elucidated based on IR, NMR of 1H- and 13C 1D, and 2D-spectroscopic techniques including DEPT, HETCOR, HMBC, and NOESY.

### 2.3. Drugs

The following drugs were used: atropine sulfate, acetylcholine hydrochloride (ACh), indomethacin, N^G^-nitro L-arginine methyl ester (L-NAME), L-phenylephrine chloride (Phe), L-arginine, ethylene glycol *bis*(*β-*aminoethylether)-N,N,N′,N′-tetraacetic acid (EGTA), caffeine, hydroxycobalamin (HDX), tetraethylammonium (TEA), and 1*H*-[1, 2, 3]oxadiazolo[4,3-*α*]quinoxalin-1-one (ODQ) (all from SIGMA). KA-acetoxy was solubilized in a mixture of distilled water cremophor at a concentration of 10 mM and diluted to the desired concentration with distilled water just before use. Indomethacin was dissolved in 0.5% w/v sodium bicarbonate. ODQ was prepared as stock solution dimethyl sulfoxide (DMSO). EGTA was added in the Ca^2+^-free Tyrode's solution. The other compounds were freely dissolved in distilled water. The final concentration of cremophor and DMSO in the bath never exceeded 0.01% was without effect when tested in control preparations (data not shown).

### 2.4. Animals

Male Wistar rats (200–300 g) were used in all experiments. Experimental protocols and procedures were approved by the Laboratório de Tecnologia Farmacêutica Animal Care and Use Committee. Animals were housed under conditions of controlled temperature ((25 ± 1)°C) and lighting (light on 6:00–18:00 h) and had access to food and tap water *ad libitum*.

### 2.5. Tissue Preparation

Rats were killed by stunning and exsanguination. The superior mesenteric artery was removed, cleaned from connective tissue and fat and sectioned in rings (1-2 mm), which were suspended by cotton threads in organ baths containing 10 mL of Tyrode's solution (composition in mM: NaCl: 158.3: KCl: 4.0; CaCl_2_: 2.0; MgCl_2_: 1.05; NaH_2_PO_4_: 0.42; NaHCO_3_: 10.0; and glucose: 5.6.), gassed with carbogenic mixture (95% O_2_ and 5% CO_2_), and maintained at 37°C for isometric *n* tension recordings. The stabilization period was of 1 h under a resting tension of 0.75 g. During this time, the solution was changed each 15 min to prevent the accumulation of metabolites. The isometric tension was recorded by a force transducer (Gould, Model GM2, USA) coupled to an amplifier recorder (Gould, USA). Endothelium was removed by gently rubbing the intimal surface of the vessels. The presence of functional endothelium was assessed by the ability of acetylcholine (ACh) (10 *μ*M) to induce more than 80% relaxation of precontracted vessels with Phe (10 *μ*M). The absence of the relaxation to ACh was taken as evidence that the vessel segments were functionally denuded of endothelium.

### 2.6. Effect of KA-Acetoxy on Superior Mesenteric Rings Precontracted with Phe or K^+^-Depolarizing Solutions (80 mM KCl)

In the first set of experiments, the ability of KA-acetoxy to cause vascular relaxation was evaluated in both endothelium-intact and endothelium-denuded mesenteric artery rings previously contracted by Phe (10 *μ*M). Under the sustained contraction elicited by Phe the vessels were exposed to cumulative concentrations of KA-acetoxy (10^−6^–1 mM).

In the second set of experiments, after the stabilization period, rings without endothelium were precontracted with KCl 80 mM on the tonic phase and different concentrations of KA-acetoxy (10^−6^–1 mM) were added cumulatively to organ bath. The extent of relaxation was expressed as the percentage of phenylephrine- or KCl-induced contraction.

### 2.7. Verification of the Participation of Endothelium-Derived Products and Muscarinic Receptors in the Relaxant Effect of KA-Acetoxy

To investigate the possible mechanism(s) responsible for KA-acetoxy induced relaxation, the preparations with endothelium were precontracted with Phe for 30 min after being early incubated with one of the following inhibitors: atropine (1 *μ*M), a nonselective antagonist of the muscarinic receptors, L-NAME (100 *μ*M), an inhibitor of the NO-synthase (NOS), L-NAME plus L-arginine (1 mM), NOS substrate, L-arginine alone, indomethacin (10 *μ*M), an inhibitor of the cyclooxygenase (COX), L-NAME + indomethacin, ODQ (10 *μ*M), selective inhibitor of the guanylyl cyclase enzyme, and hydroxocobalamine (30 *μ*M), a nitric oxide scaveng, separately.

### 2.8. Investigation of the Role of K^+^ Channels in the KA-Acetoxy-Induced Vasorelaxant Response

Phe-induced sustained contractions were obtained in endothelium-intact and endothelium-denuded mesenteric artery rings incubated with tetraethylammonium, a nonselective inhibitor of K^+^ channels (TEA, 5 mM), and then concentration-response curves to KA-acetoxy were obtained. The TEA was added 30 minutes before the contractions with Phe.

### 2.9. Effect of KA-Acetoxy on Contractions Induced by CaCl_2_

In order to access the effects of KA-acetoxy on voltage-gated Ca^2+^ channels, superior mesenteric artery rings were bathed for 15 min in Ca^2+^-free Tyrode's solution, prepared by omitting only CaCl_2_ and then exposed for an additional 15 min to a high K^+^ (60 mM) Ca^2+^-free solution. Under this new experimental condition, cumulative concentration response curves to CaCl_2_ (ranging from 1 *μ*M to 10 mM) were obtained. KA-acetoxy (3, 30, 100, 300 *μ*M, or 1 mM) was added to the preparations for 30 min, and then a new cumulative concentration-response curve for CaCl_2_ was determined. The maximal contraction obtained with the control concentration-response curve to CaCl_2_ was taken as 100% and all values were calculated as a percentage of the maximal response. Each preparation was exposed to only one diterpene-concentration. All experiments were done using endothelium-denuded superior mesenteric artery rings.

### 2.10. Effect of KA-Acetoxy on Phenylephrine- and Caffeine-Induced Contractions in Ca^2+^-Free Solution

The effect of KA-acetoxy on phenylephrine- or caffeine-sensitive calcium intracellular stores was assessed by using a protocol described by Sakata and Karaki [20]. The transient contractions were obtained in endothelium denuded rings by 10 *μ*M Phe or 20 mM caffeine in Ca^2+^-free solution before and after incubation with KA-acetoxy (100 *μ*M, 300 *μ*M or 1 mM) for 20 min. The results were expressed as percentages of the response induced by Phe or caffeine alone.

### 2.11. Statistical

In order to study the effect of KA-acetoxy on inducing relaxation, two pharmacological parameters were analysed: the *E*_max⁡_ (maximal effect generated by the agonist) and pD_2_ (−log⁡*EC*_50_). Results are expressed as means ± standard error of the mean (SEM). Student's *t*-test and one-way analysis of variance (Anova) using Bonferroni's posttest was used to analyse the data, and results were considered significant when *P* < 0.05.

## 3. Results

### 3.1. Effect of KA-Acetoxy on Superior Mesenteric Rings Precontracted with Phe- or K^+^-Depolarizing Solutions (80 mM KCl)

Table 1 shows that KA-acetoxy completely and in a concentration-dependent manner relaxed the phenylephrine induced contraction in artery segments with intact endothelium. In endothelium-denuded vessels, there was a significant rightward shift in the concentration-response curve to KA-acetoxy no change in *E*_max⁡_ as compared to the control. In rings without endothelium precontracted with K^+^-depolarizing solution (KCl 80 mM), the concentration-response curves for KA-acetoxy was significantly rightward shifted no change in *E*_max⁡_ as compared to the phenylephrine-contracted endothelium-denuded vessels (pD_2_ = 3.6 ± 0.1 and *E*_max⁡_ = 87.9 ± 4.7%).

The magnitude of contraction induced by Phe in rings with and without endothelium functional was 0.42 and 0.44 g, respectively. Similarly, the magnitude of contraction induced by KCl in rings without endothelium was 0.40 g. There were no significant differences between the magnitudes.

### 3.2. Verification of the Participation of Endothelium-Derived Products and Muscarinic Receptors in the Relaxant Effect of KA-Acetoxy

The incubation with L-NAME (100 *μ*M), indomethacin (10 *μ*M), or L-NAME (100 *μ*M) plus indomethacin (10 *μ*M) significantly shifted to the right the concentration-response curves of KA-acetoxy. In the same manner, after soluble guanylyl cyclase inhibition (ODQ 10 *μ*M), HDX (30 *μ*M), a nitric oxide scaveng, or muscarinic blockade (atropine 1 *μ*M) reduced KA-acetoxy-induced relaxation and produced a rightward displacement of the concentration-response curve for the compound. However, L-arginine (1 mM) or L-arginine plus L-NAME (100 *μ*M) had not significant effect on KA-acetoxy-induced relaxation (Table 1). The means of magnitude of contraction induced by Phe was 0.47 ± 0.10 g after incubation with the inhibitors and/or antagonist. There were no significant differences when compared with the magnitude of contraction induced by Phe in the absence of inhibitors and/or antagonist.

### 3.3. Investigation of the Role of K^+^ Channels in the KA-Acetoxy-Induced Vasorelaxant Response

In endothelium-intact and denuded rings precontracted with Phe (10 *μ*M), TEA (5 mM), a nonselective inhibitor of K^+^ channels, was not able to change KA-acetoxy relaxations (Table 2).

### 3.4. Effect of KA-Acetoxy on Contractions Induced by CaCl_2_

**Figure 2:**
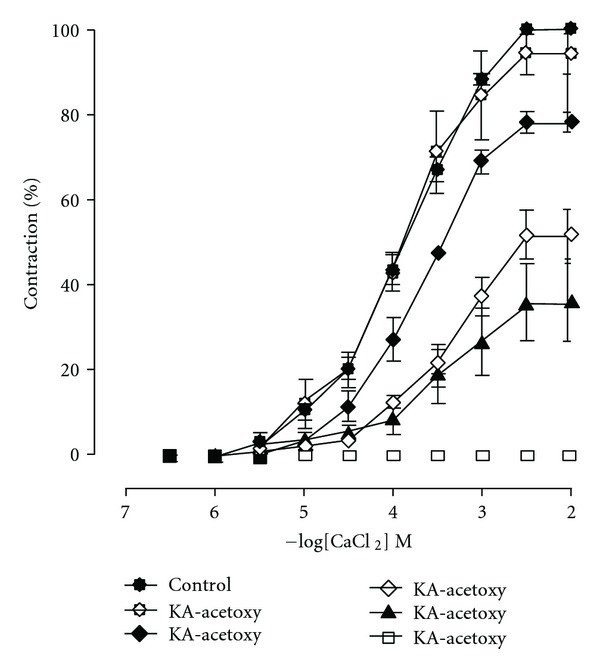
Concentration-response curves for CaCl_2_ before (• control, *n* = 12) and after the incubation of preparations with KA-acetoxy (∘ 3 *μ*M, *n* = 6), (♦ 30 *μ*M, *n* = 8), (*◊* 100 *μ*M, *n* = 6), (▲ 300 *μ*M, *n* = 5), or (□ 1 mM, *n* = 6) in rings of rat mesenteric artery without endothelium. The data were examined using one-way anova followed by the Bonferroni post-test.

Under this experimental condition, KA-acetoxy produced a nonparallel and concentration-dependent rightward shift of the CaCl_2_ concentration-response curve significantly reducing the maximal response as illustrated in Figure 2.

### 3.5. Effect of KA-Acetoxy on Phenylephrine- and Caffeine-Induced Contractions in Ca^2+^-Free Solution

**Figure 3:**
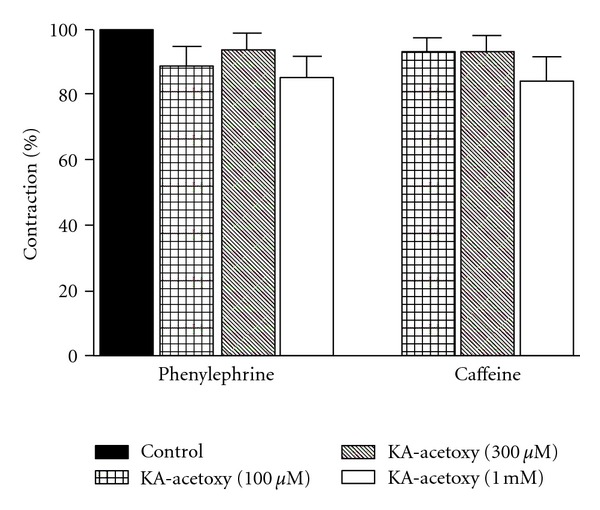
Effects of KA-acetoxy (100 *μ*M, 300 *μ*M, or 1 mM) on transient contractions induced by phenylephrine (10 *μ*M) and caffeine (20 mM) in Ca^2+^-free Tyrode's solution in isolated rat mesenteric rings without the endothelium. Values are expressed as means ± SEM of six experiments. The data were analysed by one-way anova followed by the Bonferroni post-test.

In mesenteric rings under a Ca^2+^-free solution, KA-acetoxy not inhibited transient contractions induced by 10 *μ*M phenylephrine or by 20 mM caffeine (Figure 3).

## 4. Discussion

The present work was performed in order to investigate possible vasodilator effects of the KA-acetoxy in the isolated rat superior mesenteric artery. It was observed that diterpene induced concentration-dependent vasorelaxation in the isolated rat superior mesenteric artery. The results also suggest that there are two components of the vasodilatory effect: one endothelium dependent and the other endothelium independent.

The vascular endothelium plays an important role in homeostasis by modulating vascular smooth muscle tone and acts as a main target site in hypertension and atherosclerosis. Regulation of vasodilatation by the endothelium is determined by three main components; NO, prostacyclin and endothelium derived hyperpolarizing factor (EDHF). These endothelium-derived relaxing factors diffuse to adjacent smooth muscle cells and cause them relaxation [21, 22]. To determine whether the vasodilatory effect could involve the release of NO or PGI_2_, we performed experiments with L-NAME, an inhibitor of NO synthase, and indomethacin, a potent nonselective COX inhibitor, separately. In the presence of L-NAME or indomethacin, the concentration-response curves for KA-acetoxy significantly shifted to the right with reduced *E*_max⁡_, suggesting the participation of NO and cyclooxygenase pathways in the vasorelaxant effect of KA-acetoxy.

L-arginine, a NO precursor, antagonized the effect of L-NAME, but when added alone it did not affect the relaxations induced by KA-acetoxy. The inability of this NO synthase substrate to increase the relaxation induced by KA-acetoxy may be explained in terms of there being sufficient amounts of L-arginine in the vascular endothelium. The *K*_*m*_ values of the enzyme of L-arginine are 1.5 to 2.3 *μ*M [23]. Moreover, hydroxocobalamin, a nitric oxide scaveng [24], caused a significant rightward shift of the concentration-response curve for diterpene in endothelium-intact rings, suggesting again that the action of KA-acetoxy is partially associated with the generation of NO in the vascular endothelium.

In most vascular beds, the stimulation of muscarinic receptors (M_3_ subtype) produces an intense dilation, despite the lack of vascular cholinergic innervation [25]. The muscarinic receptors responsible for relaxation are located on the endothelial cells and their stimulation leads to the release of EDRFs, mainly NO, which diffuses to adjacent smooth muscle cells and causes them to relax [21]. To determine whether KA-acetoxy-induced NO release could be secondary to the stimulation of endothelial M_3_ receptors [26], we performed experiments on intact superior mesenteric artery preparations that were early incubated with atropine. In these preparations, the vasorelaxant effect of KA-acetoxy was attenuated; in other words, the concentration-response curves for KA-acetoxy significantly shifted to the right with reduced *E*_max⁡_. These results suggest that diterpene partly could be acting via endothelial muscarinic receptor activation and consequent of participation of cGMP pathway.

It is well known that NO induces vascular smooth muscle relaxation through activation of guanylyl cyclase, leading to the accumulation of cyclic GMP. Relaxation of vascular smooth-muscle by NO-cGMP signaling involves a sequence of steps. NO released activates soluble guanylyl cyclase. This enzyme catalyzes the conversion of GPT to cGMP. cGMP-activaed protein kinase G inhibits Ca^2+^ influx, augments Ca^2+^ sequestration, and decreases the sensitivity of contractile elements to Ca^2+^ [27]. Therefore, in order to verify the participation of the NO-GMPc pathway in the effects of a KA-acetoxy in the present study, preparations were incubated with ODQ, a soluble guanylyl cyclase inhibitor. Under these conditions, the concentration-response curve to KA-acetoxy was shifted to the right with reduced *E*_max⁡_, suggesting that the relaxant response elicited by diterpene involves the NO-sGC-GMPc pathway.

EDHF plays little role in vasoactive responses of conduit vessels, it mediates a major component of the response to endothelium-dependent vasodilators in resistance arteries [28–30]. The chemical identity of EDHF is controversial, although epoxyeicosatrienoic acids, the cytochrome P-450 metabolites of arachidonic acid, have been proposed as possible candidate for EDHF [31, 32]. As mentioned, the identity of EDHF is controversial and still unknown enzyme responsible for its production. How to investigate the involvement or not of EDHF the vasorelaxant action of KA-acetoxy? Endothelium-dependent relaxation resistance to COX- and NOS-inhibitors was previously considered to be attributed to EDHF [33–35]. To test this hypothesis; we incubated the preparations with L-NAME and indomethacin, simultaneously. Under these conditions, KA-acetoxy-induced vasorelaxation was attenuated, but not abolished. Therefore, we suggest that the mechanism of the vasorelaxant action of the KA-acetoxy involves the participation of NO and PGI_2_, as well as probably the participation of EDHF. Nevertheless further experiments are needed to clear up the mechanisms involved.

The vasodilation mediated by membrane hyperpolarization is attributed to a rise in K^+^ permeability. Direct activation of K^+^ channels on arterial smooth muscle cells normally hyperpolarizes the cell membrane and thus inhibits Ca^2+^ influx through voltage sensitive Ca^2+^ channels [36]. Also, it is reported in the literature that EDRFs induce vasorelaxation by activating the K^+^ channels [37]. In addition, several natural products have been shown to induce vasorelaxant effects through the activation of K^+^ channels [38, 39]. Aiming to investigate the involvement of K^+^ channels the vasorelaxant activity elicited by diterpene, the preparations were early incubated with tetraethylammonium, a nonselective K^+^ channel blocker. This condition, the vasorelaxant activity, was not changed, suggesting that K^+^ channels are not involved in the vasorelaxant effect elicited by KA-acetoxy.

The maintenance of smooth muscle contraction depends on Ca^2+^ influx from the extracellular space through voltage- and/or receptor-operated calcium channels (VOCCs and/or receptor operated calcium channels, resp.) [40]. It is well known that the contractions to *α*_1_-adrenoceptor agonists, such as phenylephrine, are initiated by Ca^2+^ release from intracellular stores, which is followed by activation of Ca^2+^-activated channels causing depolarization of the vascular smooth muscle cell membranes and activation of voltage-gated Ca^2+^ channels [41]. Whereas that high-K^+^-induced contraction in smooth muscle is mediated by cell membrane depolarization and an increase in calcium influx through VOCCs [42]. In both cases, the major resulting effect is an increase in the intracellular calcium concentration through calcium entry.

Thus, it is proposed that the residual vasorelaxant effect of the diterpene is due to a mechanism independent of endothelium, possibly a blocking activity on the Ca^2+^ channels. Based on this assumption, we evaluated the effect of KA-acetoxy on endothelium-denuded rings precontracted with K^+^-depolarizing solutions (KCl 80 mM). KA-acetoxy was capable to inhibit contractility induced by KCl (80 mM) in endothelium-denuded mesenteric rings. This result suggests that KA-acetoxy could inhibit Ca^2+^ influx through VOCCs.

In order to strengthen the above hypothesis, KA-acetoxy was tested in the presence of CaCl_2_-induced contractions in a depolarizing medium without calcium. This protocol was based on the fact that CaCl_2_-induced contractions are elicited, almost exclusively, through Ca^2+^ influx, since the depolarization promoted by high concentrations of extracellular K^+^ induces the opening of voltage-dependent Ca^2+^ channels [43]. Under this experimental condition, KA-acetoxy produced a nonparallel and concentration-dependent rightward shift of the CaCl_2_ concentration-response curve significantly reducing the maximal response.

The release of intracellular stored Ca^2+^ is mainly regulated by IP_3_ receptor system (IP_3_Rs) and ryanodine receptor system (RyRs). The former induces Ca^2+^ release directly when the receptors are bound to IP_3_. The later may function through a Ca^2+^ induced Ca^2+^ release (CICR) mechanism when the receptors are activated by caffeine [44]. To investigate the involvement of these stores in the vasorelaxant response induced by KA-acetoxy, we performed experiments using rings contracted by phenylephrine or caffeine, in Ca^2+^-free solution, in the absence and presence of diterpene. Thus, KA-acetoxy not inhibited transient contractions induced by phenylephrine and caffeine, suggesting that the compound does not interfere in the calcium mobilization of calcium intracellular stores.

KA-acetoxy is a closely related derivate with kaurenoic cid (*ent*-kaur-16-en-19-oic acid). Similarly, KA-acetoxy was able to induce vasorelaxation vascular tissues such as kaurenoic acid. The mechanism involved in the vasorelaxant action has some similarities, such as extracellular Ca^2+^ influx blocked and activation of NO-cGMP pathway [11]. Like other diterpenes, the KA-acetoxy also induced their vasorelaxant activity by acting on multiple sites of action [11, 45, 46]. The multiple effects described here were not unlikely caused by toxicity of KA-acetoxy on vascular cells. Firstly, the relaxant effect of agent was reproducible. Secondly, the phenylephrine-induced contractile response was totally restored following 60 minutes of KA-acetoxy.

Recently, we observed that KA-acetoxy induced hypotension activity in animals with essential hypertension (Lyon hypertensive rats) (unpublished data). It is well known that small arteries, as the superior mesenteric artery, play an important role in the determination of the peripheral resistance and in the regulation of blood pressure [47]. We can suggest the hypothesis that hypotensive response could be due to a decrease in peripheral vascular resistance caused by a possible vasorelaxation, since KA-acetoxy induced vasorelaxation in superior mesenteric artery rings in normotensive rats. Therefore, the vasorelaxant activity could be used as a potential substance for antihypertensive treatment.

In addition, only substances that inhibit contractions in KCl test model are worth further examination in a search for naturally occurring calcium-antagonists. In this context, the KA-acetoxy was able to inhibit contractility induced by KCl. In this context, it is possible to suggest that the KA-acetoxy could exert antihypertensive action in vivo as other diterpenes [11, 14].

In summary, the present study demonstrated that the KA-acetoxy produced a concentration and endothelium-dependent and -independent vasorelaxation in superior mesenteric artery rings. Endothelium-dependent relaxation appears to be due to endothelial muscarinic receptors activation, NO and PGI_2_ release, as well as probably the participation of EDHF. Endothelium-independent relaxation KA-acetoxy acts through inhibition of the Ca^2+^ influx.

## Figures and Tables

**Table 1:** Comparison of *E*_max⁡_ and pD_2_ values of KA-acetoxy against tonic contractions induced by phenylephrine in isolated rat mesenteric rings.

Phenylephrine 10 (*μ*M)	*E*_max⁡_	pD_2_
(condition)	(percentage of relaxation)	(value)
Endothelium intact	92.8 ± 3.7	6.0 ± 0.3
Endothelium denuded	95.2 ± 2.8	4.6 ± 0.2***
L-NAME (100 *μ*M)	67.3 ± 4.3***	4.1 ± 0.1***
Indomethacin (10 *μ*M)	61.2 ± 7.4***	4.5 ± 0.3*
L-NAME + indomethacin	66.9 ± 3.6***	4.7 ± 0.3*
Atropine (1 *μ*M)	73.6 ± 6.6*	4.4 ± 0.2***
ODQ (10 *μ*M)	77.7 ± 3.6*	4.6 ± 0.5*
Hydroxycobalamine (10 *μ*M)	68.1 ± 6.7**	4.6 ± 0.3*
L-arginine (1000 *μ*M)	91.4 ± 2.9	5.4 ± 0.4
L-NAME + L-arginine	100 ± 0	5.3 ± 0.4

Values are expressed as means ± SEM of six experiments. These experiments were performed in mesenteric rings with functional endothelium. *P < 0.05, **P < 0.01 and ***P < 0.001 versus endothelium intact. The data were analysed by one-way Anova followed by the Bonferroni post-test.

**Table 2:** Effect of TEA in the relaxant effect of KA-acetoxy.

Phenylephrine 10 (*μ*M)	*E*_max⁡_	pD_2_
(condition)	(%) relaxation	(value)
Endothelium intact	92.8 ± 3.7	6.0 ± 0.3
Endothelium denuded	95.2 ± 2.8	4.6 ± 0.2
Endothelium intact + TEA (5 mM)	90.7 ± 3.1^a^	5.4 ± 0.4^a^
Endothelium denuded + TEA (5 mM)	100 ± 0^b^	4.4 ± 0.2^b^

Values are expressed as means ± SEM of six experiments. The data were analysed by one-way Anova followed by the Bonferroni post-test.

^a^Compared to endothelium intact.

^b^Compared to endothelium denuded.
